# Influence of light conditions on the production of chrysolaminarin using *Phaeodactylum tricornutum* in artificially illuminated photobioreactors

**DOI:** 10.1002/mbo3.1378

**Published:** 2023-09-18

**Authors:** Konstantin Frick, Tobias Ebbing, Yen‐Cheng Yeh, Ulrike Schmid‐Staiger, Günter E. M. Tovar

**Affiliations:** ^1^ Institute of Interfacial Process Engineering and Plasma Technology, Bioprocess Engineering University of Stuttgart Stuttgart Germany; ^2^ Industrial Biotechnology Fraunhofer Institute for Interfacial Engineering and Biotechnology IGB Stuttgart Germany

**Keywords:** co‐production of chrysolaminarin, fucoxanthin, and eicosapentaenoic acid, light conditions in photobioreactors, *Phaeodactylum tricornutum*, production of β‐glucans with microalgae

## Abstract

The light conditions are of utmost importance in any microalgae production process especially involving artificial illumination. This also applies to a chrysolaminarin (soluble 1,3‐β‐glucan) production process using the diatom *Phaeodactylum tricornutum*. Here we examine the influence of the amount of light per gram biomass (specific light availability) and the influence of two different biomass densities (at the same amount of light per gram biomass) on the accumulation of the storage product chrysolaminarin during nitrogen depletion in artificially illuminated flat‐panel airlift photobioreactors. Besides chrysolaminarin, other compounds (fucoxanthin, fatty acids used for energy storage [C16 fatty acids], and eicosapentaenoic acid) are regarded as well. Our results show that the time course of C‐allocation between chrysolaminarin and fatty acids, serving as storage compounds, is influenced by specific light availability and cell concentration. Furthermore, our findings demonstrate that with increasing specific light availability, the maximal chrysolaminarin content increases. However, this effect is limited. Beyond a certain specific light availability (here: 5 µmol_photons_ g_DW_^−1^ s^−1^) the maximal chrysolaminarin content no longer increases, but the rate of increase becomes faster. Furthermore, the conversion of light to chrysolaminarin is best at the beginning of nitrogen depletion. Additionally, our results show that a high biomass concentration has a negative effect on the maximal chrysolaminarin content, most likely due to the occurring self‐shading effects.

## Graphical abstract

Here we examine the influence of the amount of light per gram biomass (specific light availability) and the influence of two different biomass densities (at the same amount of light per gram biomass) on the accumulation of the storage product chrysolaminarin and potential co‐products: fucoxanthin, eicosapentaenoic acid, and fatty acids used for energy storage (C16 fatty acids), during nitrogen depletion in artificially illuminated flat panel airlift photobioreactors. Our results show that the time course of C‐allocation between chrysolaminarin and fatty acids as storage compounds depends on specific light availability and cell concentration.

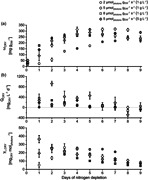

## 1 INTRODUCTION

High‐value compounds from microalgae, such as glucans, pigments, and fatty acids, have continued to attract interest in recent years, for example for the application in food, feed, and cosmetics (Gora et al., 2022; Lourenço‐Lopes et al., 2021; Neumann, Derwenskus, et al., 2018; Neumann, Louis, et al., 2018; Reis et al., 2021; Stiefvatter et al., 2021). For the production of microalgae‐derived components, the cultivation conditions are of outstanding importance (Derwenskus, 2020). During the phototrophic cultivation of microalgae, light serves as the only energy source for biomass formation and further the formation of desired compounds. Therefore, the light conditions have a great impact on autotrophic microalgae production processes. During phototrophic cultivation in closed photobioreactors, the amount of light available to a microalgae culture can be directly linked to its biomass productivity (Derwenskus, 2020; Holdmann et al., 2018). The light impinging on the surface of photobioreactors can be controlled. For example by shading when using natural light during outdoor cultivation or by dimming the light source when using artificially illuminated photobioreactors. In any case, the light available to every gram of biomass—the specific light availability (*I*_spec_)—is of special interest for the process (Holdmann et al., 2018). *I*_spec_ describes the ratio between the photon flux density (PFD) (µmol_photons_ m^−2^ s^−1^) on the surface of the photobioreactor to the total amount of biomass in the reactor volume (Holdmann et al., 2018). It not only has an impact on biomass productivity but also on the biomass composition, for example, on the accumulation of fucoxanthin (FX) in *Phaeodactylum tricornutum* (Derwenskus, 2020). Although already considered in the calculation for *I*_spec_, the culture density has a crucial impact on the light conditions in photobioreactors. With rising biomass density and despite mixing, self‐shading effects occur in microalgae cultures, affecting biomass productivity (Holdmann et al., 2018). In modern biotechnological processes, efforts should be made to use resources as efficiently as possible and not to waste them needlessly. In contrast to the outdoor production of microalgae biomass, artificially illuminated processes require electricity for light generation. Therefore, in order not to waste resources like electricity, it is of particular importance in artificially‐illuminated processes how efficiently light is converted into biomass or a desired product (light yield). Moreover, in production processes using artificial illumination, the energy costs for illumination are a major part of the operating costs of the process (Derwenskus, Weickert, et al., 2020).

Microalgae contain a multitude of different compounds and even a single strain can contain different desired compounds. Diatoms, like *P. tricornutum*, produce the carotenoid fucoxanthin, the omega‐3‐fatty acid eicosapentaenoic acid (EPA), and the 1,3‐β‐glucan chrysolaminarin (CRY) (Gao et al., 2017). Chrysolaminarin is a water‐soluble (1,3)‐(1,6)‐β‐d‐glucan, with antitumoral activity (Kusaikin et al., 2010). It promotes the health of juvenile fish (Reis et al., 2021). Furthermore, in recent studies with zebrafish, chrysolaminarin from *P. tricornutum* showed positive results against hypercholesterolemia, similar to the drug simvastatin, which is used to manage high cholesterol levels (Gora et al., 2022). Chrysolaminarin‐rich biomass also showed gut‐related benefits in a mouse study, such as an increase in short‐chain fatty acids (Stiefvatter, Neumann, et al., 2022). This makes it interesting for human nutrition as well. Further potential positive effects could already be shown in humans for example potential beneficial effects for healthy aging (Stiefvatter, Frick, et al., 2022). Chrysolaminarin closely resembles laminarin, a 1,3‐β‐glucan derived from macroalgae (Beattie et al., 1961). For laminarin, further possible applications have already been published, such as immunomodulatory properties or the promotion of animal health (Heim et al., 2015; Neyrinck et al., 2007; Sakai, 1999). If included in animal feed, it might contribute to the substitution of antibiotics in livestock farming (Lynch et al., 2010; Neyrinck et al., 2007). Laminarin also stimulates the response of vascular plants against pathogenic fungi and prevents fungal infections (Aziz et al., 2003; Cheong et al., 1991; Klarzynski et al., 2000). It can therefore be used in agriculture as well. Fucoxanthin is a xanthophyll that acts as a light‐harvesting pigment in the chloroplasts (Peng et al., 2011). Besides its antioxidative, anti‐inflammatory, and weight‐reducing properties, it shows activity against nonalcoholic fatty liver disease (NAFLD) (Fung et al., 2013; Gille et al., 2019; Heo et al., 2012; Hosokawa et al., 2004; Kotake‐Nara et al., 2001; Maeda et al., 2005, 2006; Neumann, Louis, et al., 2018; Sachindra et al., 2007). The first product against NAFLD containing fucoxanthin is already available in the U.S. (Fucovital™; Algatech). EPA is an important omega‐3 fatty acid for human nutrition and is already used as a food supplement (Ritter et al., 2013). It shows antioxidative and anti‐inflammatory effects in humans and animals (Calder, 2010; Kim & Chung, 2007). It has been published that it has a positive effect on cardiovascular diseases and high blood pressure and might prevent the development of hypertension (Connor, 2000; Frenoux et al., 2001; Kang & Leaf, 1996; Narayan et al., 2006; Prisco et al., 1998).

Chrysolaminarin serves as an energy and carbon storage compound in diatoms and is especially accumulated under nutrient‐depleted cultivation conditions, for example, nitrogen or phosphorous depletion (Frick et al., 2023; Gao et al., 2017; Kroth et al., 2008; Myklestad, 1989). Therefore, the production process for chrysolaminarin is composed of a nutrient‐replete phase (for biomass growth) and a nutrient‐depleted phase (usually nitrogen depletion) for chrysolaminarin accumulation. N‐depleted cultivation conditions are negative for the production of fucoxanthin and EPA, as it has already been reported that the fucoxanthin content as well as the EPA content are decreasing under N‐depleted conditions (Alipanah et al., 2015; Chrismadha & Borowitzka, 1994; Gao et al., 2017; Guo et al., 2016). Furthermore, the volumetric productivity of fucoxanthin and EPA is lower under N‐depleted conditions compared to nutrient‐replete conditions (Frick et al., 2023). However, even after a longer period of N‐depletion, EPA and fucoxanthin are still present in the biomass (Gao et al., 2017).

Chrysolaminarin serves as the primary energy storage product of *P. tricornutum* (Alipanah et al., 2015; Granum & Myklestad, 2002; Myklestad, 1989). Additionally, for chrysolaminarin, *P. tricornutum* accumulates triglycerides, containing specifically C16 fatty acids (C16:0 and C16:1) during nutrient depletion, which serve as energy storage as well (Yodsuwan et al., 2017). Other than fatty acids, fucoxanthin, and EPA, chrysolaminarin was not considered in most studies regarding its production with microalgae (Yang et al., 2020). Only little is published on the production of chrysolaminarin in photobioreactors using *P. tricornutum* (Frick et al., 2023; Gao et al., 2017). Moreover, the influence of different cultivation conditions on chrysolaminarin accumulation in photobioreactors has not been investigated. In experiments using *Skeletonema costatum* grown in conical flasks (250 mL) Vårum and Myklestad already found an effect of different PFDs on the accumulation of chrysolaminarin (Vårum & Myklestad, 1984). Since chrysolaminarin is produced as energy storage, it can also be expected that the loss of energy from the increased self‐shading effects that occur at higher biomass concentrations has a (negative) effect on the accumulation of chrysolaminarin. However, it is not known (and not quantified) how I_spec_ and biomass density affect the accumulation of chrysolaminarin in photobioreactors, especially in the nutrient‐depleted phase of a chrysolaminarin production process.

Here we examined the influence of the light conditions on the accumulation of chrysolaminarin during nitrogen depletion (N‐depletion) in *P. tricornutum* cultures grown in commercially available, scalable flat‐panel airlift (FPA) reactors with artificial illumination. We focused on the influence of *I*_spec_ and culture density. Besides chrysolaminarin, fatty acids were analyzed due to their role as energy and carbon storage in *P. tricornutum*. Fucoxanthin and EPA were analyzed as well, to examine the effects of the tested cultivation conditions on other potentially valuable products in the biomass. Although N‐depleted cultivation conditions are negative for the production of fucoxanthin and EPA, are both possible co‐products, which can be obtained from the produced biomass via cascaded extraction (Derwenskus, Weickert, et al., 2020; Gao et al., 2017). During the proposed phototrophic process, carbon dioxide would be fixed. Furthermore, because of the artificially illuminated closed reactor system, no surface area of (arable) land is required and the water consumption is low (Moomaw et al., 2017). Moreover, for the cultivation of the chosen organism (*P. tricornutum*), salt water can be used as the base of the cultivation media.

## 2 MATERIALS AND METHODS

### 2.1 Algae strain

All the experiments were conducted using the microalgae strain *P. tricornutum* SAG 1090‐1b. It was acquired from the Department of Experimental Phycology and Culture Collection of Algae (EPSAG) of the Georg‐August University in Göttingen, Germany.

### 2.2 Culture media

Modified Mann & Myers medium was used in all experiments. The media composition was 10 g L^−1^ NaCl, 2.4 g L^−1^ MgSO_4_ · 7 H_2_O, 0.6 g L^−1^ CaCl_2_ · 2H_2_O and 20 ml L^−1^ trace element solution. The trace element solution remained unchanged from the original recipe by Mann and Myers (Mann & Myers, 1968). Phosphorous was added separately using a 50 g L^−1^ phosphate stock solution made of potassium phosphate. The phosphate content during the experiments ranged from 20 to 200 mg L^−1^ (0.2–2.1 mmol L^−1^). Nitrogen was also added separately in the form of ammonium via a 35 g L^−1^ stock solution made of ammonium hydrogen carbonate. In the precultures, the ammonium content ranged from 30 to 300 mg L^−1^ (1.7–16.6 mmol L^−1^). During N‐depletion ammonium addition was stopped and the ammonium content in the culture medium subsequently dropped to 0 mg L^−1^. The phosphate and ammonium content of the media was analyzed via flow injection analysis (FIA) equipped with a photometric detector (see Section 2.6).

### 2.3 Illumination and light conditions

To describe the light conditions, the specific light availability (*I*_spec_) was used, as described (Holdmann et al., 2018). *I*_spec_ correlates the light intensity (PFD) on the reactor surface to the volume and density of the microalgae culture (dry biomass). For the calculation, only light in the PAR region was taken into account. *I*_spec_ was calculated according to Equation (1), with *A* = illuminated reactor surface (0.21 m^2^), PFD = photon flux density on the surface of the reactor (in µmol_photons_ m^−2^ s^−1^), *V* = culture volume (in L), *c*_DW_ = biomass concentration (in g L^−1^).

(1) $$I_{\mathrm{spec}}=\frac{A\times \mathrm{PFD}}{V\times c_{\mathrm{DW}}}.$$

Table A2 shows the light intensity (PFD) on the reactor surface during the experiments.

### 2.4 Experimental setup

To examine the influence of the light conditions, four different experimental setups were carried out, which varied in *I*_spec_ applied and/or the initial biomass concentration at the beginning of N‐depletion (see Table 1). Each experimental setup was performed in three parallel batch cultivations (biological triplicate). The three experimental cultures were inoculated from the same pre‐culture, which was grown under nutrient‐replete conditions (see Section 2.5). In the experimental cultures, the addition of ammonium was stopped and the ammonium concentration dropped to 0 mg L^−1^. The experimental cultures were cultivated for 10 days after inoculation. The first day with 0 mg L^−1^ ammonium in the medium was set as Day 0 of N‐depletion. This was usually the first day after inoculation (Setup 2, 3, 4), which is why the presented data goes up to Day 9. However, in cultures cultivated with an *I*_spec_ of 2 µmol_photons_m^−2^ s^−1^ (Setup 1), the ammonium content reached 0 mg L^−1^ three days after inoculation. Therefore, here the data presented is only from Day 0 to Day 7. During the experiments, the dry weight was measured daily (see Section 2.6) and the light intensity was adjusted to the new biomass concentration to keep *I*_spec_ constant (see Section 2.3). Moreover, each day a biomass sample for analysis was prepared from each culture (see Section 2.6).

**Table 1:** Overview of the four different experimental setups, which were carried out to examine the influence of the light conditions.

		Setup 1	Setup 2	Setup 3	Setup 4
Specific light availability (*I*_spec_)	(µmol_photons_ m^−2^ s^−1^)	2	5	8	5
Initial biomass concentration	(g L^−1^)	1	1	1	5

*Note*: Each setup was done as a biological triplicate.

The experiments were carried out in commercially available FPA reactors with a volume of 6 L (Subitec GmbH) in controlled laboratory conditions using artificial illumination. FPA reactors are modified flat plate reactors that have a special form to improve mixing. FPA reactors are mixed pneumatically by injecting an air and CO_2_ mixture through a silicone membrane at the bottom of the reactor. Cultivation conditions (pH, addition of CO_2_ to airflow, temperature, light intensity, addition of ammonium and phosphate) were monitored and controlled via a control unit (Siemens SPS). The pH was set to 7.3 in all experiments and ranged from 7.1 to 7.4. For aeration compressed air was used with an air flow of 3 L min^−1^ (0.5 v v^−1^ min^−1^). To the gas flow, pure CO_2_ was added automatically (1–20 L h^−1^, 0.5%–10% of the total airflow). The amount of added CO_2_ was controlled by the reactor control to keep the pH value in the culture media stable. Culture temperature was controlled by the reactor control via a tempered water bath in which the lower 10 cm of the reactor was submerged. The artificial illumination was done using LED panels (Nichia, NSSL157AT‐H3), which were mounted to one side of the reactor at a distance of 2 cm. The illuminated reactor surface was 0.21 m² and the microalgae cultures illuminated constantly. The LEDs emitted a light spectrum similar to sunlight (3000 K and color rendering index > 90) and could be dimmed manually using the reactor control. The light intensity (PFD) on the reactor surface during the experiments can be seen in Table A2.

### 2.5 Precultures

All experimental cultures were inoculated from precultures. These precultures were cultivated at least for 14 days in FPA reactors before the inoculation of the experimental cultures, to exclude the adaption processes of the cultures to the reactor during the experiments. For experimental cultures inoculated at 1 g L^−1^, the pre‐cultures were cultivated in a repeated fed‐batch process. As soon as the biomass concentration reached 5 g L^−1^, the pre‐cultures were diluted to 1 g L^−1^. For the experimental cultures inoculated at 5 g L^‐1^, two pre‐cultures were cultivated in two separate FPA reactors up to 8 g L^‐1^ to achieve the needed biomass concentration. Before inoculation, these two pre‐cultures were combined to establish a uniform pre‐culture.

The cultivation conditions of the pre‐cultures were similar to those of the following experiments (e.g., *I*_spec_). This excludes the ammonium content, which ranged in the pre‐cultures from 30 to 300 mg L^−1^ (1.7–16.6 mmol L^−1^). The biomass concentration was determined daily and the light was adjusted accordingly to keep *I*_spec_ constant.

### 2.6 Analytics

Culture samples were taken daily using a syringe. For the determination of the ammonium and phosphate concentration, as well as for the determination of biomass concentration, a fresh culture sample was used. For compound analysis (chrysolaminarin, fatty acids [including EPA], and fucoxanthin) the biomass in the sample was concentrated via centrifugation and washed twice to remove excess medium. Afterwards, the biomass sample was freeze‐dried. In preparation for the analysis of the compounds, cell disruption was performed using a homogenizer (Precellys24; Bertin Technologies).

#### 2.6.1 Analysis of ammonium and phosphate concentration in the culture media

The phosphate and ammonium content of the media was analyzed via FIA equipped with a photometrical detector. The method used has been previously described (Derwenskus, 2020; Holdmann et al., 2018; Münkel et al., 2013). First, a culture sample was filtered to remove any cells and cell debris. The obtained filtrate was automatically injected into a carrier stream which passed through a reaction compartment to a photometric detector. The ammonium contained in the filtrate was thereby converted to ammonia, which diffused through a gas‐permeable membrane into a solvent containing a pH indicator (bromothymol blue). The resulting color change was then detected photometrically at 620 nm. The phosphate concentration was determined using a reaction with ammonium molybdate. The resulting yellow color complex was converted to a blue color complex using ascorbic acid and detected photometrically at 880 nm. Both absorbances can be converted into the corresponding ion concentration using a calibration curve.

#### 2.6.2 Determination of biomass concentration

The method used for the determination of the biomass concentration was done as described in previous publications (Frick et al., 2023). Biomass concentration c_DW_ was determined using a pre‐dried and weighed glass‐fiber filter (pore size: 0.2 µm; MN 85/70, Macherey‐Nagel GmbH) which was placed in a Büchner funnel. The funnel was connected to a vacuum pump (MZ 2C NT; Vacuubrand GmbH). A sample of the experimental culture (5 mL) was given on the filter. Using the vacuum pump, the excess culture medium was removed. To remove traces of the remaining culture medium, 5 mL of ddH_2_O was given to the sample on the filter and removed using the vacuum pump. This washing step was carried out twice. After washing the filter with the sample was dried (MA 35; Sartorius AG) and then weighed on an analytical balance (Enteris2241‐1S; Sartorius Lab Instruments GmbH). The biomass concentration of the sample was calculated by subtracting the weight of the empty (dry) filter from the weight of the biomass‐loaded filter (dry). Biomass concentration was analyzed daily as biological triplicates.

#### 2.6.3 Determination of chrysolaminarin content

The biomass‐specific chrysolaminarin content (*ω*_CRY_) was determined via an enzymatic test kit (K‐EBHLG 08/18; Megazyme). First, chrysolaminarin was enzymatically digested into glucose molecules. Then a glucose oxidase/peroxidase reagent was added, which led to a color change based on the amount of glucose molecules present. This color change was measured photometrically and can be converted to the initial amount of chrysolaminarin in the tested sample (McCleary & Draga, 2016). The test was done using the manufacturer's instructions but scaled down by a factor of 5. This test has already been used previously for the quantification of (chryso‐)laminarin from algae (Danielson et al., 2010; Frick et al., 2023). Chrysolaminarin was analyzed daily as biological triplicates.

#### 2.6.4 Determination of fucoxanthin content

The biomass‐specific fucoxanthin content (*ω*_FX_) was analyzed with an HPLC (1200 Infinity; Agilent), according to the method by Gille et al. (2015) as previously described by (Derwenskus et al., 2019). Fucoxanthin was analyzed daily as biological triplicates.

#### 2.6.5 Analysis of fatty acids

The biomass‐specific fatty acid content (EPA [*ω*_EPA_] as well as C16 fatty acids [*ω*_C16_]) was analyzed using the transesterification method from Lepage and Roy (Lepage & Roy, 1984). The determination was done as described by (Meiser et al., 2004) via a gas chromatograph (7890A; Agilent). Fatty acids were analyzed daily as biological triplicates.

### 2.7 Calculations

#### 2.7.1 Compound concentration

The concentration of a compound x (*c*_x_ in mg L^−1^) was calculated according to Equation (2) using the biomass concentration *c*_DW_ (in g L^−1^) and the content of the compound *ω*_x_. (in mg g^−1^).

(2) $$c_{x}=c_{\mathrm{DW}}\times \omega_{x}.$$

#### 2.7.2 Volumetric productivity

The volumetric biomass productivity *Q*_DW_ (in g L^−1^ day^−1^) describes the amount of biomass produced per liter and day. It was calculated as shown in Equation (3) with *c*_DW_ = biomass concentration (in g L^−1^) at Day “*n*” (*c*_DW *n*_) and at Day “*n* − 1” (*c*_DW *n* − 1_).

(3) $$Q_{\mathrm{DW}}=c_{\mathrm{DW}\;n}-c_{\mathrm{DW}\;n-1}\;\left(g_{\mathrm{DW}}\;L^{-1}{\mathrm{day}}^{-1}\right).$$

The volumetric productivity of a compound x *Q*_X_ (in mg_x_ L^−1^ day^−1^) describes the amount of compound x produced per liter and day. It was calculated as shown in Equation (4) with *c*_x_ = concentration of the compound at Day “*n*” (*c*_x *n*_) and Day “*n* − 1” (*c*_x *n* − 1_).

(4) $$Q_{x}=c_{x\;n}-c_{x\;n-1}\;\left({\mathrm{mg}}_{x}L^{-1}{\mathrm{day}}^{-1}\right).$$

#### 2.7.3 Light yield

In artificially illuminated microalgae production processes, the conversion of light to biomass or a desired compound is of great interest, as the costs for illumination have a major economic influence (Derwenskus, Weickert, et al., 2020). The light yield (*y*_L_) describes the amount of biomass (*y*_L,DW_) or desired product x (*y*_L,x_) produced per mole photons. For *y*_L tn,_ the total amount of photons applied on the previous days until the Day “*n*” of N‐depletion was taken into account.

The light yield for biomass *y*_L,DW_ (in g_DW_ mol_photons_^−1^) was calculated as shown in Equation (5) with *c*_DW_ = biomass concentration (in g L^−1^) at Day 0 (*c*_DW *t*0_) or Day “*n*” (*c*_DW *t*n_), *V* = culture volume (in L) and *I*_*n*_ = amount of photons (in mol_photons_) applied on the reactor surface from Day 0 until Day “*n*.”

(5) $$y_{L,\mathrm{DW}\;tn}=\frac{\left(c_{\mathrm{DW}\;\mathrm{tn}}-c_{\mathrm{DW}\;t0}\right)\times V}{I_{n}}\;\left({\mathrm{mg}}_{\mathrm{DW}}\;{{\mathrm{mol}}_{\mathrm{photons}}}^{-1}\right).$$

The light yield for a compound x (*y*_L,x_ in mg_x_ mol_photons_^−1^) was calculated as shown in Equation (6) with *c*_x_ = concentration of component x (in mg_x_ L^−1^) at Day 0 (*c*_x t0_) or Day “*n*” (*c*_x *t*n_), *V* = culture volume (in L) and *I_n_* = amount of photons (in mol_photons_) applied on the reactor surface from Day 0 until the Day “*n*.”

(6) $$y_{L,x\;\mathrm{tn}}=\frac{\left(c_{x\;\mathrm{tn}}-c_{x\;t0}\right)\times V}{I_{n}}\;\left({\mathrm{mg}}_{x}\;{{\mathrm{mol}}_{\mathrm{photons}}}^{-1}\right).$$

### 2.8 Statistics

The software Matlab R2022a (MathWorks) was used to conduct the statistical analysis of the results of the experiments. Analysis of variance (ANOVA) was used to examine the statistical significance of the results. For testing the assumptions needed for ANOVA, the Jarque‐Bera test (normality, Matlab function: “jbtest”) and the Bartlett's test (equal variances, Matlab function”vartestn”) were conducted. If the assumptions for ANOVA were not met, the Kruskal–Wallis test was conducted instead of ANOVA to examine the statistical significance of the results. The Kruskal–Wallis test is similar to ANOVA but has fewer restrictions to the assumptions and is therefore recommended as the most conservative strategy when the assumptions for ANOVA cannot be assured (Sullivan et al., 2016). For ANOVA, the results are presented as *F*(*df*1, df2) and *p*, where *F* is the *F* value, *df*1 and *df*2 are the degrees of freedom and *p* is the *p* value. For the Kruskal–Wallis test, the results are presented as *χ* ^2^(*df*1, *df*2) and *p*, where *χ* ^2^ is the chi‐square, *df*1 and *df*2 are the degrees of freedom and *p* is the *p* value. We performed a *t*‐test (Matlab function “ttest”) if only two set‐ups were compared (see Table A4). The results of the *t*‐test are shown as *t*(*df*) and *p*, with “*t*” being the *t* value, “*df*” being the degree of freedom, and “*p*” being the *p* value.

Significance between groups was subsequently determined using the Tukey post hoc test if ANOVA or Kruskal–Wallis indicated a significant difference. The results of the Tukey post hoc test with *p* ≤ 0.05 are indicated in tables using lowercase letters. Significant differences (significance level *p* ≤ 0.05), analyzed via ANOVA (or Kruskal–Wallis) with Turkey post hoc test, are indicated with different letters above the values. The detailed results of the statistical tests are shown in Appendix A (see Table A5).

## 3 RESULTS

### 3.1 Effects of specific light availability on biomass production

**Figure 1:**
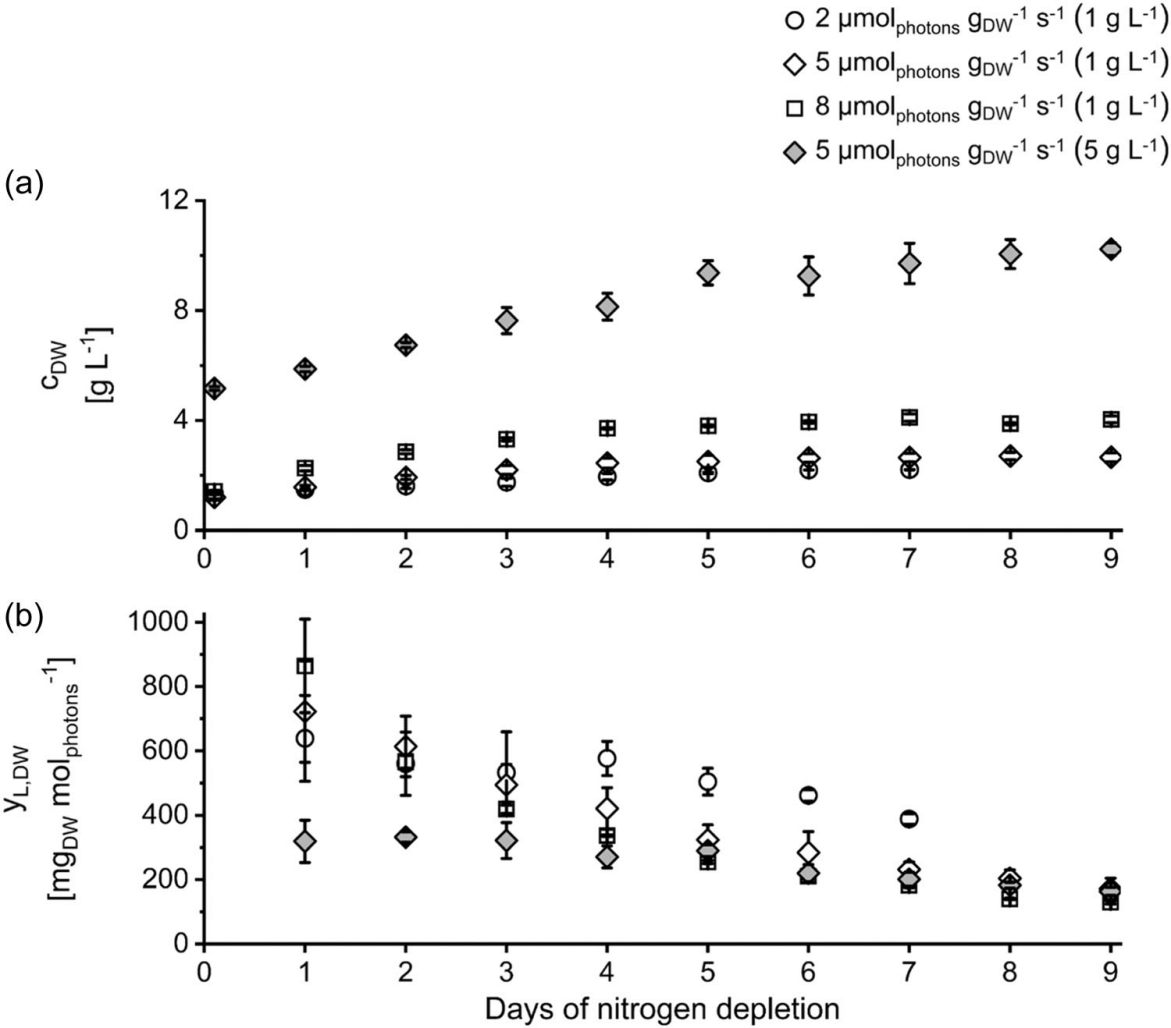
Biomass concentration *c*_DW_ (a) and the conversion of light to biomass *y*_L,DW_ (b) of *Phaeodactylum tricornutum* cultures during nitrogen‐depleted conditions grown at different specific light availabilities *I*_spec_ and inoculated with different initial biomass concentrations (in brackets). *I*_spec_ was kept constant by daily adaptation of photon flux density (see Section 2.3). ±SD, *n* = 3 analyzed as biological triplicate, see Section 2.4. Data for *c*_DW_ of setup 2 (5 µmol_photons_ m^−2^ s^−1^ and inoculated with 1 g L^−1^) previously published in Frick et al. (2023).

During N‐depletion, additional biomass was produced at every *I*_spec_ applied, which can be seen at the rising biomass concentration c_DW_ (see Figure 1). Due to the lack of nitrogen, the course of biomass production in all experimental cultures was similar to other nutrient‐depleted microalgae cultures: At the beginning of N‐depletion biomass concentration increased, but after the cells consumed their internal nitrogen storage and the accumulation of storage molecules stopped, the biomass concentration reached a plateau phase during which only minor were observed until the end of the experiments. This can especially be seen when comparing the volumetric biomass productivity *Q*_DW_ of a 3‐day time window at the beginning of N‐depletion (Days 1–3) and at the end of the experiment (Days 6–9; see Table 2). However, there was a difference in the amount of biomass produced between cultures cultivated at different *I*_spec_. At the highest tested *I*_spec_ (8 µmol_photons_ g_DW_^−1^ s^−1^) the biomass concentration increased by a factor of approximately 4 (up to 4.0 g L^−1^) compared with a factor of approximately 2 with 2 µmol_photons_ g_DW_^−1^ s^−1^ (up to 2.2 g L^−1^, see Table A1). This shows that the cultures were able to utilize the additional light at higher *I*_spec_ and convert it into biomass. That indicates that there was no photoinhibition even at cultures grown at 8 µmol_photons_ g_DW_^−1^ s^−1^. The fact that the total amount of biomass produced until the end of the experiment differed significantly shows that cultures grown at higher specific light availabilities were able to convert their internal nitrogen storage to biomass more efficiently. The ability to produce additional biomass decreased over time during N‐depletion regardless of *I*_spec_. Otherwise, all cultures should have been able to reach a comparable amount of biomass before the biomass increase stopped. Alipanah et al., who examined the response of *P. tricornutum* to nutrient depletion at a genetic level, reported that during nutrient depletion, the expression of genes associated with photosynthesis is downregulated and that the amount of chlorophyll per cell declines (Alipanah et al., 2015). The different maximally reached biomass concentrations could therefore be the result of a loss of photosynthetic capacity due to damages of the photosynthetic apparatus, which could not be compensated because of N‐depletion.

**Table 2:** Volumetric biomass productivity *Q*_DW_ at the beginning of N‐depletion (first 3 days: Days 1–3) and at the end of the experiment (last 3 days: Days 6–9) of *Phaeodactylum tricornutum* cultures grown under nitrogen‐depleted conditions with different specific light availability *I*_spec_ applied and inoculated with different initial biomass concentrations.

	Setup 1	Setup 2	Setup 3	Setup 4
Volumetric biomass productivity (*Q*_DW_) (g L^−1^ day^−1^)				
Beginning of N‐depletion (Days 1–3)	0.14 ± 0.04	0.33 ± 0.04	0.63 ± 0.03	0.82 ± 0.15
End of experiment (Days 6–9)	0.06 ± 0.02	0.01 ± 0.03	0.03 ± 0.05	0.33 ± 0.24

*Note*: Setup 1: specific light availability 2 and initial biomass concentration 1. Setup 2: specific light availability 5 and initial biomass concentration 1. Setup 3: specific light availability 8 and initial biomass concentration 1. Setup 4: specific light availability 5 and initial biomass concentration 5 (see Table 1). Each setup was done as a biological triplicate.

In the progress of N‐depletion, the conversion of light to biomass (*y*_L,DW_) decreased at every tested *I*_spec_ (see Figure 1). It decreased slowly in cultures cultivated at 2 µmol_photons_ g_DW_^−1^ s^−1^. However, at the beginning of N‐depletion *y*_L,DW_ did not differ between cultures cultivated at different *I*_spec_ (see Figure 1). Only after stagnation of biomass concentration, *y*_L,DW_ began to differ. This indicates that the microalgae were not photo‐saturated at 8 µmol_photons_ g_DW_^−1^ s^−1^, indicating that even higher *I*_spec_ can be applied during N‐depletion when cultivating *P. tricornutum* in the chosen cultivation setup. However, it has to be noted that all cultures were already adapted at the respective *I*_spec_ in the pre‐cultures before starting the experiments (see Section 2.5).

### 3.2 Effects of specific light availability on the accumulation of energy storage molecules (chrysolaminarin and C16 fatty acids)

**Figure 2:**
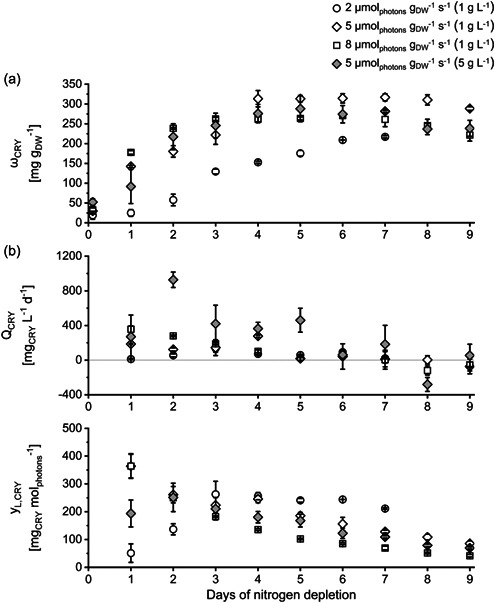
Chrysolaminarin content ω_CRY_ (a), volumetric chrysolaminarin productivity *Q*_CRY_ (b), and the conversion of light to chrysolaminarin *y*_L,DRY_ (c) of *Phaeodactylum tricornutum* cultures during nitrogen‐depleted conditions at different specific light availabilities *I*_spec_ and inoculated with different initial biomass concentrations (in brackets). *I*_spec_ was kept constant by daily adaptation of photon flux density (see Section 2.3). ±SD, *n* = 3 analyzed as biological triplicate, see Section 2.4. Data for *ω*_CRY_ of setup 2 (5 µmol_photons_ m^−2^ s^−1^ and inoculated with 1 g L^−1^) previously published in Frick et al. (2023).

**Figure 3:**
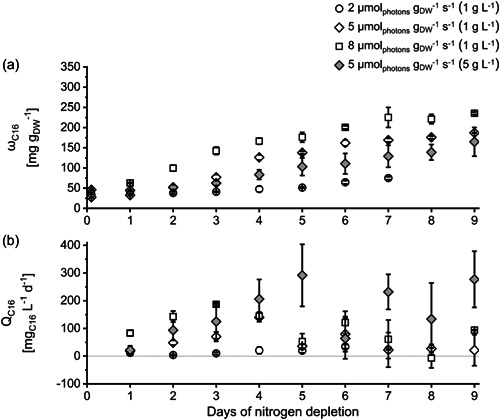
Content of C16 fatty acids *ω*_C16_ (a) and volumetric C16 fatty acid productivity *Q*_C16_ (b) of *Phaeodactylum tricornutum* cultures during nitrogen‐depleted conditions at different specific light availabilities *I*_spec_ and inoculated with different initial biomass concentrations (in brackets). *I*_spec_ was kept constant by daily adaptation of photon flux density (see Section 2.3). ±SD, *n* = 3 analyzed as biological triplicate, see Section 2.4.

The fastest initial increase in chrysolaminarin content *ω*_CRY_ was observed in cultures grown at the highest *I*_spec_ (8 µmol_photons_ g_DW_^−1^ s^−1^). However, while cultures grown at the 2 µmol_photons_ g_DW_^−1^ s^−1^ showed the lowest maximal chrysolaminarin content, the highest maximal chrysolaminarin content was observed in cultures grown at 5 µmol_photons_ g_DW_^−1^ s^−1^ (initial biomass concentration 1 g L^−1^, see Table A1). This shows that above a certain value (here 5 µmol_photons_ g_DW_^−1^ s^−1^), a higher *I*_spec_ did not result in a higher chrysolaminarin content. The reason might be the formation of other energy storage molecules (fatty acids), which require resources like energy and carbon. Chrysolaminarin is the primary energy storage product of *P. tricornutum* and the major carbon sink in the vacuole (Alipanah et al., 2015; Granum & Myklestad, 2002; Myklestad, 1989). However, fatty acids, especially C16 fatty acids (C16:0 and C16:1), also serve as energy storage in *P. tricornutum* (Yodsuwan et al., 2017). In cultures grown at 8 µmol_photons_ g_DW_^−1^ s^−1^, the C16 fatty acid content as well as the chrysolaminarin content, started to increase immediately after the beginning of N‐depletion (Day 1). Whereas in cultures grown at 5 µmol_photons_ g_DW_^−1^ s^−1^ (initial biomass concentration 1 g L^−1^), the increase of the C16 fatty acid content started later than the increase of the chrysolaminarin content (see Figures 2 and 3). It is reported that chrysolaminarin is metabolized to produce fatty acids for energy storage (like C16) after a longer period of nutrient depletion (da Costa et al., 2017; Gao et al., 2017; Li et al., 2011; Mus et al., 2013). It is proposed that the degradation of chrysolaminarin provides carbon building blocks, chemical energy (ATP), and reducing power (NADPH) needed for fatty acid formation (Alipanah et al., 2015). At higher *I*_spec_, C16 formation started earlier and thereby presumably hampered the formation of chrysolaminarin, limiting the total chrysolaminarin amount produced. However, even though a higher *I*_spec_ applied did not necessarily lead to a higher maximal chrysolaminarin content, it has to be noted that the increase of the chrysolaminarin content was faster at higher *I*_spec_. Moreover, at the beginning of N‐depletion, the volumetric chrysolaminarin productivity was also higher in cultures cultivated at higher *I*_spec_. Thus, the amount of chrysolaminarin produced in the first 3 days of N‐depletion was highest in cultures at 8 µmol_photons_ g_DW_^−1^ s^−1^. This indicates that a high *I*_spec_ could be used to shorten the time needed for chrysolaminarin accumulation in a production process.

The amount of light converted to chrysolaminarin *y*_L,CRY_ was highest at the beginning of N‐depletion in cultures grown at 5 and 8 µmol_photons_ g_DW_^−1^ s^−1^ (initial biomass concentration 1 g L^−1^) and decreased in the progress of the experiments (see Figure 2). At the beginning of the experiments, *y*_L,CRY_ did not differ between these cultures. This indicates that the metabolic pathway producing chrysolaminarin was able to use the additional energy (from the additional light) even at the highest *I*_spec_ tested (8 µmol_photons_ g_DW_^−1^ s^−1^) and has not yet reached its limit. This shows that a faster accumulation of chrysolaminarin through higher *I*_spec_ applied (as described above) did not lead to a loss of (light) energy available for the production of chrysolaminarin. In cultures grown at 2 µmol_photons_ g_DW_^−1^ s^−1^ the amount of chrysolaminarin produced per mole photons started at a lower value compared to cultures cultivated at higher *I*_spec_ and increased during the first 4 days of N‐depletion. Nevertheless, the highest amount of chrysolaminarin per mole photons was achieved at the beginning of the experiments in cultures grown at 5 or 8 µmol_photons_ g_DW_^−1^ s^−1^ (initial biomass concentration 1 g L^−1^; see Figure 2).

The content of C16 fatty acids *ω*_C16_ increased after N‐depletion, similar to chrysolaminarin, at every tested *I*_spec_ (see Figure 3). Compared with the formation of chrysolaminarin, fatty acid accumulation is more energy (ATP) and reducing power (NADPH) consuming metabolic pathway. Similar to chrysolaminarin, the C16 fatty acid content increased faster with increasing *I*_spec_ and started earlier (described above). However, in contrast to chrysolaminarin, the C16 fatty acid content differed between the experimental setups throughout the observation period and thus a higher *I*_spec_ resulted in a higher maximal C16 content (see Figure 3 and Table A1). This indicates that the surplus of energy at higher *I*_spec_ was stored as fatty acids rather than as chrysolaminarin. This also aligns with previous publications, which, as already described above, reported that chrysolaminarin is metabolized after a longer period of nutrient depletion to provide energy and carbon building blocks to synthesize fatty acids for energy storage (da Costa et al., 2017; Gao et al., 2017; Li et al., 2011; Mus et al., 2013). Therefore, the difference in biomass formation between cultures cultivated at 5 and 8 µmol_photons_ g_DW_^−1^ s^−1^ (initial biomass concentration 1 g L^−1^) as described above was (partially) due to the accumulation of fatty acids and not due to chrysolaminarin accumulation. Regarding the overall content of the here regarded energy storage molecules (chrysolaminarin and C16 fatty acids), it can be seen that the sum of chrysolaminarin and C16 fatty acid content did not exceed 500 mg g^−1^ in any setup. This indicates that it may not be possible to accumulate additional energy storage compounds beyond this content, at least not in the chosen cultivation scenario and experimental setup.

In contrast to chrysolaminarin, the volumetric productivity of C16 fatty acids *Q*_C16_ was not the highest at the beginning of the experiments but rather increased at every tested *I*_spec_ in the first days of N‐depletion. However, after it reached a maximum, the volumetric productivity of C16 fatty acids decreased in the progress of N‐depletion similar to chrysolaminarin. A higher *I*_spec_ had a positive influence on the maximal volumetric C16 productivity. Cultures grown at 8 µmol_photons_ g_DW_^−1^ s^−1^ showed a higher maximal volumetric C16 productivity than cultures grown at lower *I*_spec_ (see Figure 3 and Table A1).

### 3.3 Effects of the specific light availability on fucoxanthin and eicosapentaenoic acid during nitrogen depletion

The main focus of this paper is the production of chrysolaminarin. However, co‐products gained via cascaded extraction of the produced biomass as proposed by Derwenskus et al. may increase the economic feasibility of a potential production process (Derwenskus, Weickert, et al., 2020). Therefore, although it is reported that N‐depletion is negative for the production of fucoxanthin and EPA, both were analyzed during the experiments.

**Figure 4:**
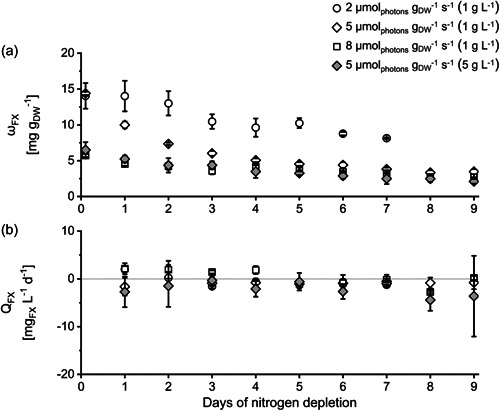
Fucoxanthin content *ω*_FX_ (a) and volumetric fucoxanthin productivity *Q*_FX_ (b) of *Phaeodactylum tricornutum* cultures during nitrogen‐depleted conditions at different specific light availabilities *I*_spec_ and inoculated with different initial biomass concentrations (in brackets). *I*_spec_ was kept constant by daily adaptation of photon flux density (see Section 2.3). ±SD, *n* = 3 analyzed as biological triplicate, see Section 2.4. Data for *ω*_FX_ of setup 2 (5 µmol_photons_ m^−2^ s^−1^ and inoculated with 1 g L^−1^) previously published in Frick et al. (2023).

It has already been reported that cultivation under N‐depleted conditions had a negative effect on fucoxanthin production and fucoxanthin content (Guo et al., 2016). Moreover, it is even reported, that a higher nitrogen concentration in the culture media promotes a higher fucoxanthin content in the biomass produced (McClure et al., 2018; Xia et al., 2013). Therefore, fucoxanthin was analyzed in our experiments only as a potential co‐product to collect data on how the different experimental setups affected the fucoxanthin content. As expected, the fucoxanthin content ω_FX_ decreased during N‐depletion in our experiments at every tested *I*_spec_ (see Figure 4), which aligns with previous publications (Alipanah et al., 2015; Derwenskus, Schäfer, et al., 2020; Huang et al., 2019). Alipanah et al. as well as Levitan et al. showed that genes related to photosynthesis were downregulated during N‐depletion, this includes genes related to the production of fucoxanthin (Alipanah et al., 2015; Levitan et al., 2015). Moreover, Levitan et al. reported that genes associated with fucoxanthin (e.g., genes related to chlorophyll a/c binding proteins) were the most downregulated in *P. tricornutum* during N‐depletion. Nevertheless, our results show that *I*_spec_ influenced the fucoxanthin content, which also affected the pre‐cultures. Cultures grown at 2 and 5 µmol_photons_ g_DW_^−1^ s^−1^ (initial biomass concentration 1 g L^−1^) had a higher fucoxanthin content at the beginning of N‐depletion compared to cultures grown at 8 µmol_photons_ g_DW_^−1^ s^−1^. It has already been described that high light intensities lead to a low fucoxanthin content, while low light intensities promote a higher fucoxanthin content in the biomass (McClure et al., 2018; Xia et al., 2013). In addition, Derwenskus already described that a lower *I*_spec_ leads to a higher fucoxanthin content in *P. tricornutum* cultures grown in FPA reactors under nutrient‐replete conditions (Derwenskus, 2020). However, Derwenskus also described, that under nutrient‐replete conditions, the volumetric fucoxanthin productivity is higher at higher *I*_spec_ as the lower fucoxanthin content can be compensated through a higher biomass productivity. Even though the volumetric fucoxanthin productivity Q_FX_, was positive in our experiments in cultures grown at 8 µmol_photons_ g_DW_^−1^ s^−1^ in the first days of N‐depletion, we could not confirm Derwenskus' observation for the cultivation under N‐depleted conditions as the volumetric fucoxanthin productivity was very low throughout the whole experiment at every tested *I*_spec_, (see Figure 4). Overall N‐depletion is not favorable for the production of fucoxanthin, as fucoxanthin productivity is far lower during N‐depletion compared to nutrient‐replete conditions (Frick et al., 2023).

**Figure 5:**
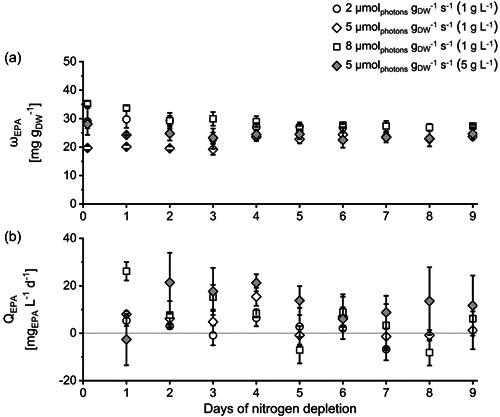
EPA content *ω*_EPA_ (a) and volumetric EPA productivity *Q*_EPA_ (b) of *Phaeodactylum tricornutum* cultures during nitrogen‐depleted conditions at different specific light availabilities *I*_spec_ and inoculated with different initial biomass concentrations (in brackets). *I*_spec_ was kept constant by daily adaptation of PFD (see Section 2.3). ±SD, *n* = 3 analyzed as biological triplicate, see Section 2.4. Data for *ω*_EPA_ of setup 2 (5 µmol_photons_ m^−2^ s^−1^ and inoculated with 1 g L^−1^) previously published in Frick et al. (2023).

EPA content *ω*_EPA_ declined during N‐depletion in cultures grown at 2 and 8 µmol_photons_ g_DW_^−1^ s^−1^. A decreasing EPA content was expected, as EPA is not used as a storage compound but serves as a membrane lipid in *P. tricornutum*. A constant or decreasing EPA content has already been reported for microalgae cultures cultivated under N‐depleted conditions (Alipanah et al., 2015; Chrismadha & Borowitzka, 1994; Gao et al., 2017). Furthermore, Levitan et al. reported that polar lipids are remobilized toward fatty acids used for energy storage during N‐depletion (Levitan et al., 2015). Previous publications also reported an EPA content for *P. tricornutum* cultivated under nutrient‐replete conditions of up to 50 mg g_DW_^−1^ (Gu et al., 2022; Steinrücken et al., 2018). Although the EPA content in our experiments is rather low compared to these previous publications, the (mostly) positive values of the volumetric EPA productivities *Q*_EPA_ observed in our experiments, show that EPA is still produced under N‐depleted conditions (see Figure 5). However, even though EPA was still produced under N‐depleted conditions, it has to be noted that the volumetric EPA productivity we observed for *P. tricornutum* under N‐depleted conditions was lower compared to the EPA productivity under nutrient‐replete conditions previously published by Derwenskus (2020). Further, Derwenskus described that under nutrient‐replete conditions, the EPA content as well as volumetric EPA productivity increases with increasing *I*_spec_ (Derwenskus, 2020). Under N‐depleted conditions, we also observed that a higher *I*_spec_ had a positive impact on the EPA content and volumetric EPA productivity, as cultures grown at 8 µmol g_DW_^−1^ s^−1^ showed the highest maximal EPA content during N‐depletion (see Figure 5 and Table A1).

### 3.4 Effects of initial biomass concentration

The general response of cultures to N‐depletion did not differ between cultures regardless of the biomass density at inoculation. In both experimental setups (inoculation at 1 and 5 g L^−1^), biomass concentration increased at the beginning of N‐depletion and slowed down after several days (see Figure 1 and Table 2), chrysolaminarin and fatty acid content increased (see Figures 2 and 3), while the fucoxanthin content declined (see Figure 4). Unsurprisingly, the volumetric productivity of biomass, chrysolaminarin, and C16 fatty acids was higher in cultures inoculated with 5 g L^−1^ compared with cultures inoculated with 1 g L^−1^. However, a higher initial biomass concentration also had negative effects on the accumulation of chrysolaminarin. Cultures inoculated with a higher biomass density showed a lower maximal chrysolaminarin content (see Figure 2 and Table A1). Furthermore, cultures inoculated with 1 g L^−1^ produced a higher amount of biomass, chrysolaminarin, and C16 fatty acids per gram initial biomass in the regarded process window (see Table A4). For microalgae cultures cultivated under nutrient‐replete conditions in FPA reactors, it has already been described that at similar *I*_spec_ biomass productivity and light yield decrease with increasing biomass density (Holdmann et al., 2018). With rising biomass concentration the self‐shading effects in the cultures are increasing, the depth of light penetration in the culture decreases and the intermixing becomes less efficient. This affects the light distribution between the cells of the culture negatively and has subsequently a negative effect on biomass productivity (Derwenskus, 2020; Holdmann et al., 2018). A higher biomass concentration influenced the light intensity experienced by the culture, as the self‐shading effects led to a loss of (light‐)energy available for biomass production (comparable to a lower light intensity.) During N‐depletion, this directly affected the formation of energy storage compounds like chrysolaminarin and fatty acids.

**Figure A1:**
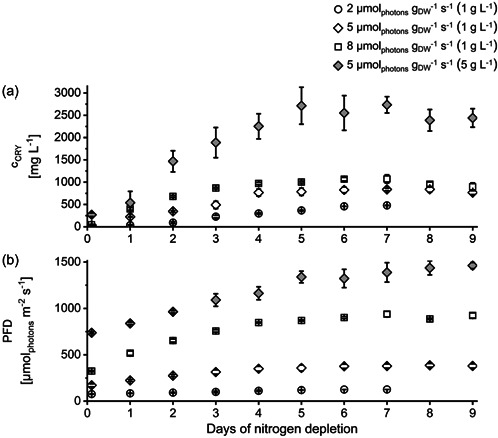
Chrysolaminarin concentration *c*_CRY_ of *Phaeodactylum tricornutum* cultures during N‐depletion (a) and photon flux density (PFD) on the reactor surface during the experiments (b). Cultures were grown under nitrogen‐depleted conditions at different specific light availabilities *I*_spec_ and inoculated with different initial biomass concentrations in brackets. *I*_spec_ was kept constant by daily adaptation of PFD (see Section 2.3). ±SD, *n* = 3 analyzed as biological triplicate, see Section 2.4.

Despite being cultivated at the same *I*_spec_, cultures inoculated with 5 g L^−1^ showed a lower fucoxanthin content at the beginning of the experiment and subsequently throughout the whole experiment, compared to cultures inoculated with 1 g L^−1^ (see Figure 4). This might be due to the culture conditions of the respective pre‐cultures (see Section 2.5). Since I_spec_ was similar, the light conditions differed in maximal intensity, as higher light intensity (PFD) on the reactor surface was required during the cultivation of cultures with higher cell density (see also Figure A1). It is reported that higher maximal light intensities lead to a decrease in fucoxanthin content (Derwenskus, 2020; Lepetit et al., 2012; Xia et al., 2013). This indicates that the cultivation at higher cell densities resulted not only in a loss of (light‐)energy due to self‐shading as described above but also led to further negative effects due to the high light intensity required. In contrast to fucoxanthin, the EPA content was not affected by a higher maximal PFD on the reactor surface, but rather by higher *I*_spec_ itself (see Figure 5) as the maximal EPA content did not differ significantly between cultures inoculated with 1 g L^−1^ and those inoculated with 5 g L^−1^ (see Table A1). The reason might be that fucoxanthin, as a light‐harvesting pigment, is more directly involved in photosynthetic processes compared with EPA, which is not as directly involved in the photosynthetic process (Boudière et al., 2014; Derwenskus, 2020; Peng et al., 2011).

### 3.5 Implications for a possible chrysolaminarin production process

The proposed production process targeting chrysolaminarin consists of two phases, as chrysolaminarin is accumulated during N‐depletion. In the first phase, biomass is produced under nutrient‐replete cultivation conditions. In this phase, the culture is cultivated under optimal culture conditions to achieve high biomass productivity in a cost/energy‐efficient process. In the second phase, the produced biomass is used to accumulate chrysolaminarin. For this, the culture is cultivated under N‐depleted cultivation conditions. In this paper, we focused on the second phase, where chrysolaminarin is accumulated. Besides chrysolaminarin, the produced biomass would contain fucoxanthin and EPA as potential co‐products. It has to be noted, that cultivation under N‐depleted conditions is not favorable for both co‐products. Fucoxanthin, as well as EPA productivity, is higher under nutrient‐replete cultivation conditions (Frick et al., 2023). The volumetric fucoxanthin productivity was negative under N‐depleted conditions in most of the experiments presented above (see Section 3.3).

Our results show that for the second (N‐depleted) phase of the chrysolaminarin production process, only a duration of up to 4 days is necessary and sensible, as *y*_L,CRY_ was highest at the beginning of the depletion and about two‐thirds of the maximal amount of chrysolaminarin was already produced after 2 days of N‐depletion (see Figures 1 and 2). Furthermore, a longer depletion phase (>4 days) did not increase the amount of chrysolaminarin produced (low volumetric chrysolaminarin productivity after Day 4; see Figure 2b) but instead led to a higher amount of C16 fatty acids (see Figure 3b). Based on our results, two different process designs for the production of chrysolaminarin from microalgae emerge.

In the first scenario, the N‐depletion phase is very short (1 or 2 days) with a high *I*_spec_ (8 or higher). This way, the high chrysolaminarin productivity and *y*_L,CRY_ at the beginning of the N‐depletion would be exploited. Due to the short N‐depletion phase, this scenario would be better suited for the co‐products of fucoxanthin and EPA, as they are mainly produced during the first (nutrient‐replete) phase of the process. N‐depletion is not favorable for the production of these two components (Alipanah et al., 2015; Huang et al., 2019). A bio‐refinery approach with the extraction of different desired products from the same biomass can increase the economic feasibility of artificially illuminated microalgae production processes (Derwenskus, Schäfer, et al., 2020; Derwenskus, Weickert, et al., 2020). However, due to the short N‐depletion phase in this scenario, the reached chrysolaminarin content would be rather low and the maximum producible amount of chrysolaminarin would not be reached as well. However, a short N‐depletion phase might also enable a consecutive process design, where at the end of the N‐depletion chrysolaminarin‐rich biomass is partially harvested and the remaining biomass, replenished with nutrients, is used as inoculum for the following growth phase. This approach would be similar to a process Benvenuti et al. examined for the production of fatty acids used for energy storage (TAGs) with the microalgae *Nannochloropsis* sp. (Benvenuti et al., 2016). They found that the cultures showed a lower TAG content during their semi‐continuous process compared to a batch process. However, the biomass productivity was higher in the semi‐continuous process, so the overall TAG productivity was similar between the semicontinuous and the batch process. As TAGs are used for energy storage similar to chrysolaminarin, a semicontinuous should also be investigated for the production of chrysolaminarin.

In the second scenario, the N‐depletion phase would be 3 or 4 days to ensure that a high proportion of the producible amount of chrysolaminarin is achieved. The longer N‐depletion would also lead to a higher chrysolaminarin content, which is beneficial in the downstream processing. In this scenario, the culture density could be higher, as *y*_L,CRY_ was similar in our experiments if the N‐depletion lasted longer than 2 days. Therefore, a higher volumetric chrysolaminarin concentration could be achieved, which would benefit the space‐time yield of the process (see Figure A1). However, this scenario would focus mainly on the production of chrysolaminarin, as the longer N‐depletion is negative for the co‐products fucoxanthin and EPA (see above). To work properly, at least two separate photobioreactors would be needed for this process design, one for biomass production and another for chrysolaminarin accumulation.

In the end, the overall economics of the production process would determine the choice between the two described scenarios as well as the layout of the final process. Here the prices of the different products have to be included. Therefore, a techno‐economic analysis would be required to identify the most profitable layout of the process.

## 4 CONCLUSION

In conclusion, light is an important factor in a possible microalgae production process involving chrysolaminarin production. Our results showed that for a chrysolaminarin production process, only a N‐depletion phase of up to 4 days is needed and sensible because the maximal amount of chrysolaminarin was accumulated in the first days of N‐depletion. The depletion phase can be even shortened with higher *I*_spec_. Although a higher *I*_spec_ fastened chrysolaminarin accumulation, it did not necessarily increase the maximal chrysolaminarin content. Above a certain *I*_spec_ (here 5 µmol_photons_ g_DW_^−1^ s^−1^) the maximal chrysolaminarin content did not increase further. A higher initial biomass concentration led to a lower maximal chrysolaminarin content, indicating that self‐shading effects were of relevance here.

## AUTHOR CONTRIBUTIONS

**Konstantin Frick**: Conceptualization (lead); data curation (equal); formal analysis (supporting); funding acquisition (supporting); investigation (lead); methodology (equal); project administration (supporting); validation (lead); visualization (lead); writing—original draft (lead); writing—review and editing (equal). **Tobias Ebbing**: Methodology (equal); visualization (supporting); writing—original draft (supporting); writing—review and editing (equal). **Yen‐Cheng Yeh**: Data curation (equal); formal analysis (lead); writing—original draft (supporting); writing—review and editing (equal). **Ulrike Schmid‐Staiger**: Conceptualization (supporting); funding acquisition (equal); methodology (equal); project administration (equal); resources (equal); supervision (equal); writing—original draft (supporting); writing—review and editing (equal). **Günter E. M. Tovar**: Funding acquisition (equal); project administration (equal); resources (equal); supervision (equal); writing—original draft (supporting); writing—review and editing (equal).

## CONFLICT OF INTEREST STATEMENT

None declared.

## ETHICS STATEMENT

None required.

## Data Availability

All data generated or analyzed during this study are included in this published article and its appendix A.

## APPENDIX A

See Figure A1 and Tables A1, A2, A3, A4, A5, A6

**Table A1:** Maximal volumetric productivity of chrysolaminarin *Q*_CRY_, fucoxanthin *Q*_FX_, EPA *Q*_EPA_, C16‐fatty acids *Q*_C16,_ and biomass *Q*_DW_, final biomass concentration *c*_DW_, and maximal content of chrysolaminarin *ω*_CRY_, fucoxanthin *ω*_FX_, EPA *ω*_EPA_ and C16‐fatty acids *ω*_C16_ of *Phaeodactylum tricornutum* cultures grown under nitrogen depleted conditions with different specific light availability *I*_spec_ applied and inoculated with different initial biomass concentrations.

		*I*_spec_ = 2	*I*_spec_ = 5	*I*_spec_ = 8	*I*_spec_ = 5
		DW_*t*0_ = 1	DW_*t*0_ = 1	DW_*t*0_ = 1	DW_*t*0_ = 5
Max. *Q*_DW_	(g L^−1^ day^−1^)	0.2 ± 0.0^a^ Day 4	0.4 ± 0.1^a^ Day 1	0.8 ± 0.1^b^ Day 1	1.2 ± 0.1^c^ Day 5
*c*_DW_ (final)	(g L^−1^)	2.2 ± 0.0^a^ Day 7	2.7 ± 0.2^a^ Day 9	4.0 ± 0.1^b^ Day 9	10.2 ± 0.2* Day 9
Max. *Q*_CRY_	(mg L^−1^ day^−1^)	135 ± 46^a^ Day 3	277 ± 11^ab^ Day 4	356 ± 39^b^ Day 1	927 ± 89^c^ Day 2
Max. *ω*_CRY_	(mg g^−1^)	217 ± 3^a^ Day 7	317 ± 9^a^ Day 7	271 ± 7^a^ Day 6	288 ± 33^a^ Day 5
Max. *Q*_C16_	(mg L^−1^ day^−1^)	35 ± 7^a^ Day 6	140 ± 16^ab^ Day 4	187 ± 6^ab^ Day 3	292 ± 112^b^ Day 5
Max. *ω*_C16_	(mg g^−1^)	75 ± 4^a^ Day 7	187 ± 1^ab^ Day 9	235 ± 3^b^ Day 9	165 ± 36^ab^ Day 9
Max. *Q*_FX_	(mg L^−1^ day^−1^)	2.0 ± 0.6^ab^ Day 1	−0.5 ± 0.6^a^ Day 7	2.1 ± 1.1 ^b^ Day 1	−0.3 ± 0.8^ab^ Day 3
Max. *ω*_FX_	(mg g^−1^)	14.0 ± 1.8^a^ Day 0	14.4 ± 0.2^a^ Day 0	5.7 ± 0.2^a^ Day 0	6.5 ± 1.1^a^ Day 0
Max. *Q*_EPA_	(mg L^−1^ day^−1^)	6 ± 4^a^ Day 4	15 ± 4^ab^ Day 4	26 ± 4^bc^ Day 1	31 ± 7^c^ Day 2
Max. *ω*_EPA_	(mg g^−1^)	30 ± 3^ab^ Day 1	24 ± 3^a^ Day 6	35 ± 1^b^ Day 0	28 ± 1^a^ Day 0

*Note*: *I*_spec_ was kept constant by daily adaptation of PFD (see Section 2.3). ±SD, *n* = 3 analyzed as biological triplicate, see Section 2.4. Data for *c*_DW_, *ω*_CRY_, *ω*_FX_, *ω*_EPA_ of setup 2 (5 µmol_photons_ m^−2^ s^−1^ and inoculated with 1 g L^−1^) previously published in Frick et al. (2023).

* This value was excluded from the statistical analysis.

**Table A2:** Photon flux density on the reactor surface during the experiments.

	Values shown in µmol_photons_ m^−2^ s^−1^			
	*I*_spec_ = 2	*I*_spec_ = 5	*I*_spec_ = 8	*I*_spec_ = 5
Day	DW_*t*0_ = 1	DW_*t*0_ = 1	DW_*t*0_ = 1	DW_*t*0_ = 5
0	76 ± 2	171 ± 15	324 ± 8	737 ± 9
1	85 ± 2	223 ± 10	517 ± 23	839 ± 13
2	92 ± 5	275 ± 11	653 ± 16	963 ± 12
3	100 ± 8	314 ± 23	757 ± 13	1090 ± 68
4	111 ± 7	350 ± 26	847 ± 6	1163 ± 70
5	119 ± 1	358 ± 32	869 ± 8	1339 ± 63
6	125 ± 1	376 ± 22	901 ± 11	1322 ± 99
7	126 ± 1	378 ± 22	938 ± 30	1388 ± 105
8	‐	386 ± 19	887 ± 9	1437 ± 76
9	‐	380 ± 23	923 ± 28	1463 ± 33

*Note*: *I*_spec_ was kept constant by daily adaptation of PFD (see Section 2.3).

**Table A3:** Fucoxanthin concentration at the beginning of N‐depletion and at the end of the experiments of *Phaeodactylum tricornutum* cultures grown under nitrogen‐depleted conditions with different specific light availability *I*_spec_ applied and inoculated with different initial biomass concentrations.

	*I*_spec_ = 2	*I*_spec_ = 5	*I*_spec_ = 8	*I*_spec_ = 5
	DW_*t*0_ = 1	DW_*t*0_ = 1	DW_*t*0_ = 1	DW_*t*0_ = 5
*c*_FX_ (mg L^−1^)				
Start (Day 0)	19 ± 3	17 ± 2	8 ± 0	34 ± 5
End (Day 9 or 7)	18 ± 0	9 ± 2	11 ± 0	22 ± 3

*Note*: *I*_spec_ was kept constant by daily adaptation of PFD (see Section 2.3). ±SD, *n* = 3 analyzed as biological triplicate, see Section 2.4.

**Table A4:** Biomass‐specific biomass productivity *P*_DW_, biomass‐specific chrysolaminarin productivity *P*_CRY_, and biomass‐specific productivity of C16 fatty acids *P*_C16_ in the regarded process window from Day 0 to Day 9 (whole experimental duration).

		*I*_spec_ = 5	*I*_spec_ = 5
		DW_*t*0_ = 1	DW_*t*0_ = 5
*P*_DW_	(g_DW_ g_*t*0_^−1^)	1.2 ± 0.3 ^a^	1.0 ± 0.0 ^a^
*P*_CRY_	(mg_CRY_ g_*t*0_^−1^)	617 ± 90 ^a^	420 ± 38 ^b^
*P*_C16_	(mg_C16_ g_*t*0_^−1^)	391 ± 53 ^a^	279 ± 66 ^a^

*Note*: Biomass‐specific productivities were calculated according to Equations (A1) and (A2). Cultures were grown at a specific light availability (*I*_spec_) of 2, 5, or 8 µmol_photons_ g_DW_^−1^ s^−1^. Cultures were inoculated with an initial biomass concentration (DW_t0_) of 1 or 5 g L^−1^. *I*_spec_ was kept constant by daily adaptation of PFD (see Section 2.3). ±SD, *n* = 3 analyzed as biological triplicate, see Section 2.4.

**Table A5:** Statistical results of ANOVA and Kruskal–Wallis with Tukey post‐hoc test for all experiments.

	ANOVA		Tukey post hoc test					
	*F* value(*df*1, *df*2)	*p* Value	S1 to S2	S1 to S3	S1 to S4	S2 to S3	S2 to S4	S3 to S4
Max. *Q*_DW_	*F*(3, 8) = 71.98	3.95 **×** 10^−6^	2.45 **×** 10^−1^	1.71 **×** 10^−4^	4.86 **×** 10^−6^	1.27 **×** 10^−3^	1.77 **×** 10^−5^	4.56 **×** 10^−3^
*c*_DW_ (final)	*F*(2, 5) = 96.40	1.01 **×** 10^−4^	5.70 **×** 10^−2^	1.26 **×** 10^−4^	‐	2.89 **×** 10^−4^	‐	‐
Max. *Q*_CRY_	*F*(3, 8) = 83.30	2.25 **×** 10^−6^	1.12 **×** 10^−1^	1.46 **×** 10^−2^	2.11 **×** 10^−6^	4.98 **×** 10^−1^	9.63 **×** 10^−6^	2.56 **×** 10^−5^
Max. *Q*_EPA_	*F*(3, 8) = 11.20	3.09 **×** 10^−3^	2.94 **×** 10^−1^	1.27 **×** 10^−2^	3.25 **×** 10^−3^	1.80 **×** 10^−1^	3.90 **×** 10^−2^	7.04 **×** 10^−1^
Max. *Q*_FX_	*F*(3, 8) = 6.37	1.63 **×** 10^−2^	5.50 **×** 10^−2^	9.99 **×** 10^−1^	7.73 **×** 10^−2^	4.51 **×** 10^−2^	9.95 **×** 10^−1^	6.33 **×** 10^−2^
Max. *ω*_EPA_	*F*(3, 8) = 8.69	6.73 **×** 10^−3^	1.40 **×** 10^−1^	1.27 **×** 10^−1^	8.61 **×** 10^−1^	4.56 **×** 10^−3^	3.88 **×** 10^−1^	4.23 **×** 10^−2^

	Kruskal–Wallis test		Tukey post hoc test					
	*χ* ^2^(*df*1, *df*2)	*p* Value	S1 to S2	S1 to S3	S1 to S4	S2 to S3	S2 to S4	S3 to S4
Max. *Q*_C16_	*χ* ^2^(3, 7) = 9.41	2.43 **×** 10^−2^	8.42 **×** 10^−1^	2.65 **×** 10^−1^	2.57 **×** 10^−2^	6.85 **×** 10^−1^	1.19 **×** 10^−1^	6.85 **×** 10^−1^
Max. *ω*_CRY_	*χ* ^2^(3, 7) = 7.11	6.86 **×** 10^−2^	4.76 **×** 10^−2^	6.55 **×** 10^−1^	3.20 **×** 10^−1^	3.78 **×** 10^−1^	7.58 **×** 10^−1^	9.27 **×** 10^−1^
Max. *ω*_FX_	*χ* ^2^(3, 8) = 8.45	3.61 **×** 10^−2^	9.87 **×** 10^−1^	8.14 **×** 10^−2^	1.74 **×** 10^−1^	1.74 **×** 10^−1^	3.24 **×** 10^−1^	9.87 **×** 10^−1^
Max. *ω*_C16_	*χ* ^2^(3, 7) = 8.32	3.99 **×** 10^−2^	4.46 **×** 10^−1^	2.57 **×** 10^−2^	6.55 **×** 10^−1^	4.51 **×** 10^−1^	9.83 **×** 10^−1^	2.52 **×** 10^−1^

**Table A6:** Statistical results from *t*‐test.

	*t*‐test	
	*t* (*df*)	*p* Value
*P*_DW_	*t*(2) = 1.30	3.23 **×** 10^−1^
*P*_CRY_	*t*(2) = 4.70	4.23 **×** 10^−2^
*P*_C16_	*t*(2) = 3.14	8.80 **×** 10^−2^

To compare cultures inoculated with 1 and 5 g L^−1^, the biomass‐specific productivity *P* was calculated for biomass as well as for chrysolaminarin as well as C16 fatty acids. The biomass‐specific productivity was calculated over the whole experiment (regarded process window Day 0 to Day 9. The biomass‐specific productivity *P*_DW_ was calculated as shown in Equation (A1) with *c*_DW_ = biomass concentration (in g L^−1^) at Day 0 (*c*_DW *t*0_) and Day 9 (*c*_DW *t*9_).

(A1)
$$P_{\mathrm{DW}}=\frac{\left(c_{\mathrm{DW}\;t9}-c_{\mathrm{DW}\;t0}\right)}{c_{\mathrm{DW}\;t0}}\;\left(g_{\mathrm{DW}}\;\;{g_{\mathrm{DW}\;\;t0}}^{‐1}\right).$$

The biomass‐specific chrysolaminarin (or C16 fatty acid) productivity *P*_X_ was calculated as shown in Equation (A2) with *c*_DW_ = biomass concentration (in g L^−1^) at Day 0 (*c*_DW *t*0_) and *c*_x_ = concentration of chrysolaminarin (or C16 fatty acids) (in mg L^−1^) at Day 0 (*c*_x *t*0_) or Day 9 (*c*_x *t*9_).

(A2)
$$P_{x}=\frac{\left(c_{x\;t9}-c_{x\;t0}\right)}{c_{\mathrm{DW}\;t0}}\;\left({\mathrm{mg}}_{x}\;\;{g_{\mathrm{DW}\;t0}}^{‐1}\right).$$
